# RETRACTED: Bilyy et al. Rapid Generation of Coronaviral Immunity Using Recombinant Peptide Modified Nanodiamonds. *Pathogens* 2021, *10*, 861

**DOI:** 10.3390/pathogens14111184

**Published:** 2025-11-19

**Authors:** Rostyslav Bilyy, Quentin Pagneux, Nathan François, Galyna Bila, Roman Grytsko, Yuri Lebedin, Alexandre Barras, Jean Dubuisson, Sandrine Belouzard, Karin Séron, Rabah Boukherroub, Sabine Szunerits

**Affiliations:** 1 Danylo Halytsky Lviv National Medical University, Pekarska Str., 69, 79010 Lviv, Ukraine; halyna.bila@gmail.com (G.B.); grytskoroman@gmail.com (R.G.); 2 University of Lille, CNRS, Centrale Lille, University Polytechnique Hauts-de-France, UMR 8520-IEMN, F-59000 Lille, France; quentin.pagneux@univ-lille.fr (Q.P.); alexandre.barras@univ-lille.fr (A.B.);; 3 U1019-UMR 9017-CIIL-Center for Infection and Immunity of Lille, Institut Pasteur de Lille, University of Lille, CNRS, INSERM, CHU Lille, F-59000 Lille, France; nathan.francois@ibl.cnrs.fr (N.F.); jean.dubuisson@ibl.cnrs.fr (J.D.); sandrine.belouzard@ibl.cnrs.fr (S.B.); karin.seron@ibl.cnrs.fr (K.S.); 4 Xema Co., Ltd., Akademika Efremova Str., 23, 03179 Kyiv, Ukraine; lebedin@xema.com.ua

The Journal retracts the article “Rapid Generation of Coronaviral Immunity Using Recombinant Peptide Modified Nanodiamonds” [1], as cited above.

Following the publication of a correction to this article [2], further concerns were brought to the attention of the Editorial Office regarding the integrity of a number of images presented in this publication [1].

Adhering to our standard procedure, the Editorial Office and Editorial Board conducted an investigation that confirmed an overlap between Figure 2A published in [1] and Figure 2B published in [3], as well as between Figure 6C in [1] and Figure 5 published in [4] later corrected in [5]; and identified overlap between Figure S2D published in [1] and Figure 2B in [6] previously published by a similar authorship group, being the same material however. While the authors fully collaborated within this process, supporting material and explanations provided did not meet Editorial Board expectations in order to resolve the issues raised. As a result, the Editorial Board has lost confidence in the integrity of the findings and decided to retract this publication [1], as per MDPI’s retraction policy (https://www.mdpi.com/ethics#_bookmark30).

This retraction was approved by the Editor-in-Chief of the journal *Pathogens*.

Dr. Jean Dubuisson, Dr. Sandrine Belouzard, and Dr. Karin Séron agree to this retraction. Dr. Rostyslav Bilyy and Dr. Sabine Szunerits disagree with this retraction. The remaining authors did not provide a comment on this decision.

## References

[1] Bilyy R., Pagneux Q., François N., Bila G., Grytsko R., Lebedin Y., Barras A., Dubuisson J., Belouzard S., Séron K. (2021). RETRACTED: Rapid Generation of Coronaviral Immunity Using Recombinant Peptide Modified Nanodiamonds. Pathogens.

[2] Bilyy R., Pagneux Q., François N., Bila G., Grytsko R., Lebedin Y., Barras A., Dubuisson J., Belouzard S., Séron K. (2023). Correction: Bilyy et al. Rapid Generation of Coronaviral Immunity Using Recombinant Peptide Modified Nanodiamonds. *Pathogens* **2021**, *10*, 861. Pathogens.

[3] Turcheniuk V., Raks V., Issa R., Cooper I.R., Cragg P.J., Jijie R., Dumitrascu N., Mikhalovska L.I., Barras A., Zaitsev V. (2015). Antimicrobial activity of menthol modified nanodiamond particles. Diam. Relat. Mater..

[4] Paryzhak S.Y., Dumych T.I., Peshkova S.M., Bila E.E., Lutsyk A.D., Barras A., Bilyy R., Szunerits S., Boukherroub R. (2019). Interaction of 4 allotropic modifications of carbon nanoparticles with living tissues. Ukr. Biochem. J..

[5] Paryzhak S.Y., Dumych T.I., Peshkova S.M., Bila E.E., Lutsyk A.D., Barras A., Bilyy R., Szunerits S., Boukherroub R. (2025). Corrigendum: Interaction of 4 allotropic modifications of carbon nanoparticles with living tissues. Ukr. Biochem. J..

[6] Łoczechin A., Séron K., Barras A., Giovanelli E., Belouzard S., Chen Y.T., Metzler-Nolte N., Boukherroub R., Dubuisson J., Szunerits S. (2019). Functional carbon quantum dots as medical countermeasures to human coronavirus. ACS Appl. Mater. Interfaces.

